# Mediating effects of depression on anxiety and leisure constraints in patients with breast cancer

**DOI:** 10.1186/s12905-019-0838-7

**Published:** 2019-11-20

**Authors:** Hsiu-Mei Huang, Jun-Hung Lai, Tsai-Wei Huang

**Affiliations:** 1 Department of Nursing, Ditmanson Medical Foundation Chia-Yi Christian Hospital, Chia-Yi, Taiwan; 2 Department of Internal Medicine, Erlin Christian Hospital, ChangHua, Taiwan; 3 School of Nursing, College of Nursing, Taipei Medical University, Taipei, Taiwan; 4 Cochrane Taiwan, Taipei Medical University, Taipei, Taiwan

**Keywords:** Breast Cancer, Depression, Anxiety, Leisure constraints

## Abstract

**Background:**

Patients with breast cancer often exhibit high levels of anxiety and depression and a considerable decrease in their ability to participate in leisure activities, which result in the long-term disruption of their daily lives. This study intended to explore the relationships among anxiety, leisure constraints, and depression and evaluate whether depression mediates the effects of anxiety on leisure constraints in patients with breast cancer.

**Method:**

This prospective study included 106 patients with breast cancer. All the patients completed the Taiwanese version of the Hospital Anxiety and Depression Scale and Leisure constraints questionnaire. Path analysis was used to test the mediating role of depression.

**Results:**

Leisure constraints, anxiety, and depression were positively interrelated and co-occurred in the patients. The accelerated bootstrapping confidence intervals of the indirect effect did not include zero (0.276–1.663). Moreover, depression completely mediated the effects of anxiety on leisure constraints in patients with earlier cancer stages but not in patients with advanced cancer stages.

**Conclusions:**

Depression is a crucial mechanism underlying the relationship between anxiety and leisure constraints in patients with breast cancer. Although many patients experience minimal disruption of activities and roles during survivorship, they are unable to perform functional activities and satisfactorily play their roles. This is the first study to explore leisure constraints in patients with breast cancer and investigate the mediating role of depression that underlies the relationship between anxiety and leisure constraints. The current findings are clinically crucial because they suggest the need to consider the simultaneous management of anxiety and depression for alleviating leisure constraints.

## Background

Breast cancer is a major health threat and cause of mortality in women worldwide [1]. In Taiwan, breast cancer is the most common cancer in women and a major cause of cancer-related deaths [2]. The survival rates of patients with breast cancer have increased in recent years [3, 4]. Psychological distress is particularly high in patients with cancer, and it adversely affects their quality of life as well as survival and recurrence rates. Patients often report high levels of anxiety and depression [5]. Nearly 50% of women with early-stage breast cancer presented the symptoms of severe depression or anxiety, or both in the first year after diagnosis [6]. Another study showed that 36.7% of the patients with early-stage breast cancer experienced mood disorders (9.6, 27.1, and 8.6% exhibited severe depression, mild depression, and anxiety, respectively) [7]. Moreover, young women who had received a new diagnosis of metastatic breast cancer experienced anxiety symptoms, although depression was less common [8]. In particular, depression has been associated with poor breast cancer survival [9, 10].

According to a systematic review, there was a strong association between insufficient physical activity and a decreased prognosis in patients with breast cancer [11]. Furthermore, physical activity interventions may alleviate psychological problems by exerting small-to-moderate beneficial effects on emotional distress and anxiety [12]. Leisure-time physical activities have a wide range of physical and mental health benefits [13, 14]. Encouraging patients to perform activities and continue leisure activities may reduce health care expenditure [15]. However, information regarding leisure constraints in patients with breast cancer and the evidence of relationships among anxiety, depression, and leisure constraints are scant.

Leisure constraint model presented by Crawford and Godbey (1987) [16] and Crawford, Jackson, and Godbey (1991) [17] have been widely adopted as crucial topics in studies on leisure behavior. This model had identified 3 major sources for leisure constraints: structural, interpersonal, and intrapersonal [18]. Structural constraints, such as financial resources, availability of facilities, availability of time, and climate, are factors that affect leisure preferences, choices, and actual participation. Interpersonal constraints are factors that affect the formation of relationships between individuals, such as failure to find suitable co-participants. Intrapersonal constraints reflect psychological states and personal characteristics such as stress, anxiety, depression, and socialization activities. In non-Western environments, few studies have investigated leisure constraints, particularly in patients with cancer [19]. So far, research on leisure constraints is still in its infancy.

Restoring the normal life and leisure activities of patients with cancer is necessary. However, psychological distress may adversely affect these patients’ abilities to participate in leisure activities. Therefore, we intended to explore the relationships among leisure constraints, anxiety, and depression and investigated whether depression mediates the effects of anxiety on leisure constraint. The study hypothesized that anxiety increases the severity of leisure constraints by causing depression in patients with breast cancer.

## Methods

### Study design and setting

A convenience sample of women with breast cancer was recruited at a local hospital in central Taiwan. Participants selection criteria were as follows: 1) age older than 20 years; 2) had undergone breast cancer surgery, including breast-conserving surgery (BCS), modified radical mastectomy (MRM), or MRM combined with immediate breast reconstruction (IBR); 3) could communicate in Mandarin or Taiwanese. This study was approved by an institutional review board [CYCH-IRB 102060]. Written consents were obtained from the participants.

### Measure

A semi-structured interview tool to collect the patients’ demographic data (age, marital status, education level, income, disease information) and type of treatment received. Also, each participant completed the Hospital Anxiety and Depression Scale (HADS) and the Leisure Constraints Questionnaires (LCQ) [Additional file 1].

### HADS scores

The HADS is a well-established screening instrument for depression and anxiety in patients with cancer [20, 21]. The HADS has been extensively used in study populations in oncology research, and it is a reliable screening measure in this sensitive patient population [22]. It is a 14-item self-reporting instrument, 2 subscales of 7 items each, namely HADS-Depression and HADS-Anxiety, each of which uses a 4-point scale (0: no problems to 3: maximum distress). Thus, scores on each subscale range from 0 to 21. High scores indicate relatively severe symptoms; the severity of symptoms is interpreted as no (0–7), mild (8–10), moderate (11–14), and high (15–21).

### Leisure constraints questionnaire

Leisure constraints are “factors that are assumed by researchers or perceived or experienced by individuals that limit the formation of leisure preferences or inhibit or prohibit participation and enjoyment in leisure” [23]. In this study, a questionnaire, which was designed to measure the perceived leisure constraints in patients with breast cancer, was developed. Based on the research of Crawford (1991) [17], it assessed 3 discrete types of constraints (intrapersonal, interpersonal, and structural constraints) and measured the severity of each type of constraint. A 5-point Likert rating scale for measuring leisure constraints (from 1 = completely disagree to 5 = fully agree) was used to assess the consistency of each of the 20 items. Principal component exploratory factor analysis with the varimax rotation method was used to determine the presence of distinct constraint dimensions in the patients’ responses. Originally, 20 items were used to represent 3 types of constraints. Because these 3 dimensions were expected to be almost equally crucial, multiple variables with high loading on each factor were used to enhance the interpretability of results [24]. Items with an eigenvalue of > 1 and a factor loading of at least .50 were selected for each factor. The reliability of factor dimensions was calculated using the reliability procedure of the SPSS software. The Cronbach method was used to examine the internal consistency of the leisure constraints questionnaire, with the standard value of > 0.70 reflecting satisfactory internal consistency [25]. The 3 factors were defined as follows: “intrapersonal constraints” (α = .95, 9 items), “interpersonal constraints” (α = .92, 6 items), and “structural constraints” (α = .88, 5 items). The findings verified the importance of all 3 distinct constraint dimensions. To examine further research issues, we used these 3 factors of perceived leisure constraints. The Chinese version of leisure constraints questionnaire is available upon request.

### Procedure

One of our team members described the study purpose to the patients, obtained their consents, and approached those who met the selection criteria independently. The recruited patients were asked to fill in the questionnaires by themselves. For those who were not capable to carry out the task, the researcher read out the questions and recorded the answers provided by the patients. Also, the researcher was onsite to answer any question raised during the questionnaire administration.

### Statistical analyses

**Fig. 1:**
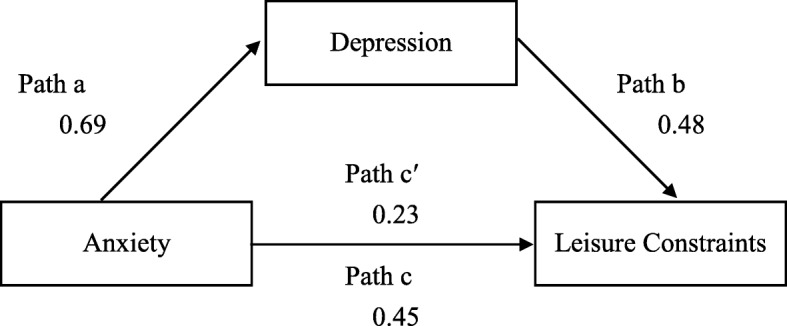
Testing the Mediating Effect on Leisure Constraints

A path coefficient analysis by using multiple regression analysis was conducted to examine the path proposed by Baron and Kenny [26]. We hypothesized that depression mediates the relationship between anxiety and leisure constraints in patients with breast cancer. In the first equation, the mediator (depression) was regressed on the independent variable (anxiety). In the second equation, the dependent variable (leisure constraint) was regressed on the independent variable (anxiety). In the third equation, the dependent variable (leisure constraint) was regressed on the mediator (depression) and the independent variable (anxiety). In addition to these 3 regression equations verifying the relationship among the mediation models, the bootstrapping method was used to examine the reliability of mediating effects [27]. The PROCESS macro for SPSS (version 22) which is specifically written for mediation, moderation, and conditional process analysis, was used to perform mediation analysis [28]. Roles of variables (i.e., independent variable, dependent variable, mediator, moderator, covariate) were provided to the macro and it estimates all the path coefficients, standard errors, t- and *p*-values, confidence intervals, and other statistics. Further explanations on statistical concepts are described in a recent article published by the authors [29]. The attribution of the independent variable to the dependent variable sustained when the regression model was significant. The hypothesized directions of attribution are shown in Fig. 1. Using 5000 bootstrapped samples generated, the mediating effect was verified when the bootstrapped 95% confidence intervals of the indirect effect did not include zero.

## Results

### Patient characteristics

In total, 106 patients with breast cancer participated in this study (the response rate was 96.3%). The demographic characteristics of the patients are listed in Table 1. The disease information of the patients was as follows: 68.9% were at tumor stage 1 to 2, 49.1% patients had undergone MRM and 43.4% had undergone BCS, and 78.3% were receiving chemotherapy at the time of the study.

**Table 1:** Characteristics of the Study Patients (*n* = 106)

Variables	n (%)	Mean (SD), Range
Age (years)		52.48 (9.17), 25–74
Marital status		
Married	87 (82.1)	
Unmarried	8 (7.5)	
Others	11 (10.4)	
Religion		
No	10 (9.4)	
Yes	96 (90.6)	
Tumor stage		
Stage 0	10 (9.4)	
Stage 1–2	73 (68.9)	
Stage 3–4	23 (21.7)	
Surgery		
BCS	46 (43.4)	
MRM	52 (49.1)	
MRM + IBR	8 (7.5)	
Chemotherapy received until now		
No	23 (21.7)	
Yes	83 (78.3)	
Radiation received until now		
No	43 (40.6)	
Yes	63 (59.4)	
Hormone therapy received until now		
No	52 (49.1)	
Yes	54 (50.9)	

### Descriptive statistics for leisure constraints, anxiety, and depression

The mean [SD] score of the HADS-anxiety was 5.74 [3.82] (range 0–15), and the proportions of the patients with mild, moderate, and severe anxiety were 62.3, 26.4, and 11.3%, respectively. The mean [SD] score of HADS-depression was 5.36 [3.52] (range 0–17), and the proportions of the patients with mild, moderate, and severe depression were 77.4, 12.2, and 10.4%, respectively. The factor loadings of each of the 3 factors measured using the leisure constraints questionnaire are presented in Table 2. The average scores divided by the number of items in intrapersonal, interpersonal, and structural constraints were 2.61, 2.29, and 2.62, respectively; thus, structural constraints were the most crucial factor. The global leisure constraint score was 50.06 [16.32], indicating that this constraint exerted an effect of “moderate” intensity on the patients with breast cancer (Table 3).

**Table 2:** Leisure Constraints Dimension from Factor Analysis

Factors/Items	Factor 1	Factor 2	Factor 3
	Intrapersonal	Interpersonal	Structural
17. Unstable health condition	.840		
18. I am so unwell	.832		
20. Fear of infection	.777		
16. Fear of increasing burden on others	.751		
19. Physical appearance change	.738		
11. Physical symptoms	.722		
14. Cannot relieve symptoms	.707		
9. Lack of entertainment information	.571		
10. No time	.562		
1. It is not necessary		.815	
3. Personality		.814	
4. Lack of experience and skills		.743	
2. Fear of being injured during activities		.739	
13. No one is engaged with me		.614	
12. No family support		.576	
15. No sufficient financial support			.555
8. Lack of transportation			.501
5. Not enough space and facilities			.829
6. Not suitable leisure facilities			.827
7. Leisure places are too crowded			.654
Eigenvalue	11.963	1.426	1.088
Variance Explained	59.817	7.131	5.438
Cumulative Variance	59.817	66.948	72.386
Scale Mean Score	2.61	2.29	2.62

**Table 3:** Summary of Questionnaire Responses of the Patients with Breast Cancer (*n* = 106)

Variables	n (%)	Mean (SD)	Range	α
HADS-Anxiety		5.74 (3.82)	0–15	.86
Mild (0–7 points)	66 (62.3)			
Moderate (8–10 points)	28 (26.4)			
Severe (11–21 points)	12 (11.3)			
HADS- Depression		5.36 (3.52)	0–17	.76
Mild (0–7 points)	82 (77.4)			
Moderate (8–10 points)	13 (12.2)			
Severe (11–21 points)	11 (10.4)			
LCQ-Leisure constraints		50.06 (16.32)		.96
Intrapersonal constraints (9 items)		23.45 (8.13)		.94
Interpersonal constraints (6 items)		13.75 (4.59)		.90
Structural constraints (5 items)		13.09 (4.60)		.92

### Mediating effects of depression on anxiety and leisure constraints

To examine if cancer staging plays a role in the mediating effects, we conducted the analyses in all patients, patients with earlier cancer stages (stage 0, 1, or 2), and patients with advanced cancer stages (stage 3 or 4).

The results for all patients are summarized in Table 4. The first step was to determine whether the presumed mediator (depression) and predictor (anxiety) were associated. The results indicated that anxiety was significantly associated with depression (β = 0.69, *P* < 001). The second step involved regressing the outcome variable (leisure constraints) on the presumed mediator (depression). Depression was significantly associated with leisure constraints (β = 0.48, *P* < 001). Testing for mediation was the third step, and it involved regressing the outcome variable (leisure constraints) on the predictor (anxiety). Anxiety was significantly associated with leisure constraints (β = 0.45, *P* < .001). The final step involved regressing leisure constraints on both the predictor (anxiety) and the presumed mediator (depression). The strength of each relationship is shown in Fig. 1. The predictor-outcome relationship must be not significant and the mediator-outcome relationship must be significant to sustain a complete mediation model. When depression was controlled, the previously significant relationship between the predictor (anxiety) and the outcome (leisure constraints) became non-significant (β = 0.23, *P* = .051). On the other hand, the mediator-outcome relationship was significant. These met the mediation theoretical requirement. The accelerated bootstrapping confidence intervals of the indirect effect did not include zero (0.276 to 1.663), indicating that the model was reliable and that depression completely mediated the effects of anxiety on leisure constraints.

**Table 4:** Mediating Effect of Depression between Anxiety and Leisure Constraints in All Cancer Patients (*n* = 106)

Testing steps in mediation model		t	B	β	R^2^	*P*-value
Testing step 1 (path a)						
Predictor	to Mediator	9.78	0.77	0.69	0.48	< .0001
Testing step 2 (path b)						
Mediator	to Outcome	5.60	2.29	0.48	0.23	< .0001
Testing step 3 (path c) ^*k*^						
Predictor	to Outcome	5.19	0.11	0.45	0.20	< .0001
Testing step 4 (path c′) ^*k*^						
Predictor	to Outcome	1.97	0.99	0.23	0.26	.051
Mediator		2.73	1.53	0.32		.007

ß estimates represent the regression coefficient

The results for subjects with earlier cancer stage are summarized in Table 5. Testing step 1 indicated that anxiety was significantly associated with depression (β = 0.61, *P* < 001) and testing step 2 indicated that depression was significantly associated with leisure constraints (β = 0.46, *P* < 001). Testing for mediation at the third step indicated that anxiety was significantly associated with leisure constraints (β = 0.43, *P* < 001). The final step involved regressing leisure constraints on both anxiety and depression. When depression was controlled, the previously significant relationship between the anxiety and leisure constraints became non-significant (β = 0.24, *P* = 051). On the other hand, the mediator-outcome relationship was significant. The accelerated bootstrapping confidence intervals of the indirect effect did not include zero (0.234 to 1.595), indicating that the model was reliable and that depression completely mediated the effects of anxiety on leisure constraints.

**Table 5:** Mediating Effect of Depression between Anxiety and Leisure Constraints in Early Cancer Stages (*n* = 83)

Testing steps in mediation model		t	B	β	R^2^	*P*-value
Testing step 1 (path a)						
Predictor	to Mediator	6.89	0.52	0.61	0.37	< .0001
Testing step 2 (path b)						
Mediator	to Outcome	4.60	2.48	0.46	0.21	< .0001
Testing step 3 (path c) ^*k*^						
Predictor	to Outcome	4.28	2.00	0.43	0.18	< .0001
Testing step 4 (path c′) ^*k*^						
Predictor	to Outcome	1.98	1.13	0.24	0.24	.051
Mediator		2.51	1.68	0.31		.014

ß estimates represent the regression coefficient

The results for subjects with advanced cancer stages are summarized in Table 6. Testing step 1 indicated that anxiety was significantly associated with depression (β = 0.88, *P* < 001) and testing step 2 indicated that depression was significantly associated with leisure constraints (β = 0.60, *P* = .002). Testing for mediation at the third step indicated that anxiety was significantly associated with leisure constraints (β = 0.59, *P* = 0.003). At the final step, when depression was controlled, the previously significant relationship between the anxiety and leisure constraints became non-significant (β = 0.26, *P* = 0.50). In contrast with previous analyses, the mediator-outcome relationship was not significant. The accelerated bootstrapping confidence intervals of the indirect effect included zero (−0.729 to 3.971), indicating that the model was non-reliable.

**Table 6:** Mediating Effect of Depression between Anxiety and Leisure Constraints in Advanced Cancer Stages (*n* = 23)

Testing steps in mediation model		t	B	β	R^2^	*P*-value
Testing step 1 (path a)						
Predictor	to Mediator	8.57	0.86	0.88	0.78	< .0001
Testing step 2 (path b)						
Mediator	to Outcome	3.44	1.88	0.60	0.36	.002
Testing step 3 (path c) ^*k*^						
Predictor	to Outcome	3.31	1.79	0.59	0.34	.003
Testing step 4 (path c′) ^*k*^						
Predictor	to Outcome	0.68	0.78	0.26	0.37	.50
Mediator		1.00	1.18	0.38		.33

ß estimates represent the regression coefficient

## Discussion

To our knowledge, this is the first study that investigates leisure constraints in patients with breast cancer. Treatment of diseases is now possible owing to advancements in medical technology; however, patients with cancer require treatment as well as an improvement in their quality of life. Maintaining regular leisure habits and promoting physical activity are part of the pursuit of improving the quality of life among patients with cancer. However, patients with cancer encounter physical and psychological distress, which may lead to the loss of self-care ability and confidence in the pursuit of leisure. Patients with breast cancer experience multiple symptoms associated with the disease and its treatment. Two commonly observed psychology symptoms (anxiety and depression) and leisure constraints in patients with breast cancer were selected for analysis in this study. Notably, this study demonstrated that anxiety, leisure constraints, and depression are interrelated in the patients with breast cancer and that depression mediates the relationship between anxiety and leisure constraints. Furthermore, we found that depression completely mediated the effects of anxiety on leisure constraints in patients with earlier cancer stages but not in patients with advanced cancer stages.

Anxiety is the most common psychological distress in patients with breast cancer [30, 31]. Although the high prevalence of anxiety symptoms in patients with breast cancer is known, the influence of anxiety disorders on cancer prognosis have been relatively less studied compared with the impact of depression. In studies on patients with chronic diseases, anxiety disorders were closely associated with increased health care use, decreased physical well-being, and increased physical disability [32]. In addition, the mechanism underlying anxiety or depression should be considered among the toxic side effects of relevant treatments. Patients may experience difficulties in participating in physical activities [8].

Inactivity adversely affects the symptoms and survival rate of patients with cancer. Psychology, accessibility, time, partners, and security factors can all become crucial leisure constraints [33]. Stressors may influence anxiety through its effect on satisfaction with leisure activities [34]. Regular leisure activities can mitigate stress and depression [35]. In this study, intrapersonal constraints involved “individual psychological states and attributes;” the primary intrapersonal constraints were unstable health condition, fear of increasing burden on others, changes in physical appearance, and the effects of symptoms. Interpersonal constraints were “the result of interpersonal interactions or relationships between individuals’ characteristics;” the chief interpersonal constraints were a patient’s personality, absence of the need for leisure, lack of family support, and lack of friends who are engaged in similar activities. Clearly, social relationships played a major role in shaping leisure activities. Family relationships impinged on some people’s freedom to participate in activities, but the lack of relationships prevented others from engaging in activities that they previously enjoyed [18]. Structural constraints were “intervening factors between leisure preference and participation;” the major structural constraints were insufficient financial support, lack of transportation, and unsuitable leisure facilities. Constraints that were assumed to play an intervening role in the leisure preference–participation relationship were considered “only one of the ways in which barriers may be associated with preferences and participation” [16]. They also noted that these constraints might be interrelated. Leisure benefits were derived from spiritual experience, spiritual well-being, and spiritual coping with stress. A study reported leisure factors that produce spiritual benefits [36]. Consistent with the findings of other studies [19, 33, 37], we also found that structural constraints were the most crucial constraints, followed by intrapersonal and interpersonal constraints. The item “no time for leisure” usually belongs to structural constraints; however, in the present study, this item was placed under intrapersonal constraints. Hence, the patients with breast cancer did not actually have insufficient time, but rather perceived that they had insufficient time. This indicates that leisure constraints in patients with breast cancer in this study were affected by psychological factors. The illness might have bright down their spirit to interpret self and the world in a negative way which is common across populations [38].

The symptoms of breast cancer experienced during treatment affect many women and their partners as they attempt to resume functional activities and crucial life roles during early survivorship. Disruptions in these valued activities and roles may affect the health and well-being of patients and their partners during and after early survivorship [39]. Successful participation in meaningful activities and roles can significantly improve health benefits, including a decrease in stress and anxiety levels. Conversely, strategies that effectively reduce anxiety and depression can also reduce leisure constraints in patients with breast cancer and increase their motivation to participate in leisure activities. The long-term consequences of activity limitations and role restrictions have been associated with serious health concerns, including physiological changes, depression, and anxiety, which lead to chronic diseases and poor quality of life [40, 41]. The present study showed that anxiety can further increase leisure constraints through depression. We should develop interventions to resolve psychological distress so that patients can resume leisure activities more effectively and improve their quality of life as soon as possible.

This study has a few limitations. First, because of the cross-sectional design of the study, changes in symptoms over time were not investigated. Replications and additional longitudinal studies that focus on changes in relationships among these symptoms over time are warranted. Certainly, it is possible that those with high depression and anxiety levels might had already perceived that their leisure time was limited even before they had a cancer diagnosis. The role of a such preexisting psychiatric symptoms require further investigation. Second, the current mediation modeling approach did not concern possible confounders. Newer research into the areas of mediation and moderation [42] or alternative models such as the Rubin’s causal model may be used to establish the possible mediation and confounding paths [43] and brought forth a more modern understanding of those phenomena [42]. Third, this study enrolled patients with breast cancer who were likely to be in a relatively stable condition. Therefore, seriously ill patients were not represented in this study, and the results of this study may not be generalizable to that population. Moreover, this study was limited by its small sample size. Additional studies may need to be replicated using a larger sample. Any conclusion from this study may be tentative; nevertheless, these results may provide useful insights for future research.

## Conclusions

This study demonstrated that anxiety, leisure constraints, and depression are interrelated in the patients with breast cancer and that depression completely mediates the relationship between anxiety and leisure constraints in patients with earlier cancer stages but not in patients with advanced cancer stages. The current findings are clinically crucial because they indicate the need to consider the simultaneous management of patient anxiety and depression, because the simultaneous resolution of the symptoms of emotional distress may alleviate leisure constraints while enhancing the willingness to participate in leisure activities.

## Supplementary information

**Additional file 1.** Leisure constraints questionnaire. English translation of the Chinese version of leisure constraints questionnaire. The Chinese version is available upon request.

## Data Availability

The data sets used and analyzed during the current study are available from the corresponding author on reasonable request.

## Abbreviations

BCS: Breast-conserving surgery
HADS: Hospital Anxiety and Depression Scale
IBR: Immediate breast reconstruction
LCQ: Leisure Constraints Questionnaires
MRM: Modified radical mastectomy
