# Supplementary material for: Mediating effects of depression on anxiety and leisure constraints in patients with breast cancer
Source: BMC Womens Health. 2019 Nov 20;19:141. doi: 10.1186/s12905-019-0838-7 (PMC6868872; doi:10.1186/s12905-019-0838-7)
Supplement: Supplementary file 1 — Additional file 1. Leisure constraints questionnaire. English translation of the Chinese version of leisure constraints questionnaire. The Chinese version is available upon request. [file 12905_2019_838_MOESM1_ESM.docx]

Leisure constraints questionnaire (English version)

**What are your opinions on the barriers of leisure?**

This is a questionnaire inquiring your leisure barriers. Please read each question carefully and choose appropriate an answer which is closest your barring reasons.

|  | Very much disagree | Do not agree | Fair | agree | Very much agree |
| --- | --- | --- | --- | --- | --- |
| 1. I do not want to engage in leisure activity because it is unnecessary. |  |  |  |  |  |
| 2. I do not want to engage in leisure activity because I am afraid of injury during activity. |  |  |  |  |  |
| 3. I do not want to engage in leisure activity because of my personality. |  |  |  |  |  |
| 4. I do not want to engage in leisure activity because of the lack of experience and skills. |  |  |  |  |  |
| 5. I do not want to engage in leisure activity because there are insufficient of places and facilities. |  |  |  |  |  |
| 6. I cannot engage in leisure activity because there are no suitable leisure facilities. |  |  |  |  |  |
| 7. I do not want to engage in leisure activity because the leisure places are crowded. |  |  |  |  |  |
| 8. I cannot engage in leisure activity because I lack transportation tools. |  |  |  |  |  |
| 9. I cannot engage in leisure activity because of the lack of leisure information. |  |  |  |  |  |
| 10. I cannot engage in leisure activity because of the lack of time. |  |  |  |  |  |
| 11. I do not want to engage in leisure activity because of my physical symptoms. |  |  |  |  |  |
| 12. I cannot engage in leisure activity because of the lack of support from my family. |  |  |  |  |  |
| 13. I do not want to engage in leisure activity because no one goes with me. |  |  |  |  |  |
|  | Very much disagree | Do not agree | Fair | agree | Very much agree |
| 14. I cannot engage in leisure activity because I could not find a way to relieve the symptoms. |  |  |  |  |  |
| 15. I do not want to engage in leisure activity because I do not have enough money. |  |  |  |  |  |
| 16. I do not want to engage in leisure activity because afraid of adding extra burden on others. |  |  |  |  |  |
| 17. I do not want to engage in leisure activity because my health condition is unstable. |  |  |  |  |  |
| 18. I do not want to engage in leisure activity because I am sick and too tired. |  |  |  |  |  |
| 19. I do not want to engage in leisure activity because of the change of my body appearance. |  |  |  |  |  |
| 20. I do not want to engage in leisure activity because of the fear of infection. |  |  |  |  |  |
